# An Appreciation

**Published:** 1913-08

**Authors:** John Wesley Long

**Affiliations:** Emeritus Professor Diseases of Women and Children, Medical College of Virginia, Founder Fellow Southern Surgical and Gynecological Association; President Guilford County Medical Society, Etc., Greensboro, North Carolina


TEXAS MEDICAL JOURNAL.

Established July, 1885

F. E. DANIEL, M. D., - - - - Editor, Publisher and Proprietor

Published Monthly.—Subscription, $1.00 a Year.

VOL. XXIX. AUSTIN, AUGUST, 1913. No. 2.

The publisher is not responsible for the views of the contributors.

### Original Articles.

For Texas Medical Journal.

### An Appreciation.

JOHN ALLAN WYETH, M. D., LL. D., THE SURGEON, THE BUILDER, THE TEACHER.

BY JOHN WESLEY LONG, M. D.,
Emeritus Professor Diseases of Women and Children, Medical College of Virginia,: Founder Fellow Southern Surgical and Gynecological Association; President Guilford County Medical Society, Etc., Greensboro, North Carolina.

I enjoyed to the fullest my attendance on the recent Clinical Congress of American Surgeons, which was held in New York City. I went early and stayed late, and only regretted my impossibility to see it all. America has much to be proud of, but there is nothing that reflects more credit on her glorious history than her surgeons and their achievements. It is a revelation to see and hear and watch the great surgeons of our country work. Knowing many of them personally, as I do from repeated visits to their clinics, it gave me an increased zest for the occasion of the Congress.

In addition to our own distinguished surgeons, we had Mr. Lane from London, famous for his bone work, a specimen of which I saw, and it was superb; also, his Lane's kink in the "ileum" and his work on "intestinal stasis." Dr. Weibel, who is Wertheim's assistant, and is said to be better than Wertheim himself, was present as an invited guest, and operated.

But of all the men I saw in New York taking part in the clinics there was none who impressed me more than did my old friend and teacher, Dr. John A. Wyeth. This is not to be marveled at, since in common with hundreds of surgeons throughout the country I feel that Dr. Wyeth is in a sense the beau-ideal of what a surgeon should be.

He is the founder of the first post-graduate school in America, and has devoted his life largely to the promotion and development of post-graduate studies. The Polyclinic stands as an enduring monument to his fame, and shall ever be an evidence of his labors. When I first attended the old Polyclinic down on Thirty-fourth Street, in 1887, I thought then it was, par excellence, the greatest school of its kind within my knowledge. When, the other day, I was shown by Dr. Wyeth over the magnificent twelve-story building, costing $800,000, at Fiftieth Street, with all its wonderful equipment, I realized with joy that it was second to none. While other men have labored with Dr. Wyeth in the building of the Polyclinic, and from them no word of praise shall be withheld, yet the Polyclinic is Dr. Wyeth's life work—materialized. When he said as he stood upon its roof garden, overlooking the surrounding city, "I have realized my dream," I thought I caught a faint glimpse of what he meant.

But the structure of steel and stone, with its equipment, is the least part of the Polyclinic. It is the men who are made—developed—grown—there that makes the Polyclinic what it is. I saw Dr. Wyeth with paternal solicitude showing a young surgeon how to do a difficult operation. The great teacher turned to the audience and said, "This is one of the glories of my life, to take the young fellow—the raw material—and make a great man out of him."

I do not wish to appear fullsome, but I do want to offer my flowers while the recipient can see and smell and appreciate them. This may Wyeth is a great man—great in his accomplishments; great in his human sympathies; honored and beloved by the profession, and the laity as well, but most of all by those who know him best.

I say three cheers for the Southern surgeon who as a lad of 18 followed Forrest through the fortunes of war; the founder of the first post-graduate school in America; the man who in the very center and rush of our greatest city has overcome every difficulty: and even yet, while approaching the time of the sere and yellow leaf, stands out pre-eminently in the forefront as the finest type of American surgeon!

---

A Wise Prescription.—Doctor: How is your appetite? Patient: Fine! I can eat anything. Doctor: Well, don't for a while and you will get better.

